# Dynamic Myocardial Perfusion CT for the Detection of Hemodynamically Significant Coronary Artery Disease

**DOI:** 10.1016/j.jcmg.2021.07.021

**Published:** 2021-09-15

**Authors:** Fay M.A. Nous, Tobias Geisler, Mariusz B.P. Kruk, Hatem Alkadhi, Kakuya Kitagawa, Rozemarijn Vliegenthart, Michaela M. Hell, Jörg Hausleiter, Patricia K. Nguyen, Ricardo P.J. Budde, Konstantin Nikolaou, Cezary Kepka, Robert Manka, Hajime Sakuma, Sachin B. Malik, Adriaan Coenen, Felix Zijlstra, Ernst Klotz, Pim van der Harst, Christoph Artzner, Admir Dedic, Francesca Pugliese, Fabian Bamberg, Koen Nieman

**Affiliations:** a Department of Radiology and Nuclear Medicine, Erasmus University Medical Center, University Medical Center Rotterdam, Rotterdam, the Netherlands;; b Department of Cardiology, Erasmus University Medical Center, University Medical Center Rotterdam, Rotterdam, the Netherlands;; c Department of Cardiology, University of Tuebingen, Tuebingen, Germany;; d Coronary Disease and Structural Heart Diseases Department, Institute of Cardiology, Warsaw, Poland;; e Institute of Diagnostic and Interventional Radiology, University Hospital Zurich, University of Zurich, Zurich, Switzerland;; f Department of Advanced Diagnostic Imaging, Mie University Graduate School of Medicine, Tsu, Japan;; g Department of Radiology, University Medical Center Groningen, University of Groningen, Groningen, the Netherlands;; h Department of Cardiology, Faculty of Medicine, Friedrich-Alexander-Universität Erlangen-Nürnberg, Erlangen, Germany;; i Department of Cardiology, Ludwig-Maximilians University, Munich, Germany;; j Veterans Affairs Palo Alto Healthcare System, Cardiology Section, Palo Alto, California, USA;; k Stanford University, Division of Cardiovascular Medicine, Stanford, California, USA;; l Stanford Cardiovascular Institute, Stanford, California, USA;; m Department of Radiology, University Hospital of Tübingen, Tübingen, Germany;; n Department of Cardiology, University Heart Center and Institute of Diagnostic and Interventional Radiology, University Hospital Zurich, University of Zurich, Zurich, Switzerland;; o Department of Radiology, Mie University Graduate School of Medicine, Tsu, Japan;; p Veterans Affairs Palo Alto Healthcare System, Thoracic and Cardiovascular Imaging Section, Palo Alto, California, USA;; q Stanford University, Division of Cardiovascular Imaging (Affiliated), Stanford, California, USA;; r Siemens Healthineers, Forcheim, Germany;; s Department of Cardiology, University Medical Center Groningen, University of Groningen, Groningen, the Netherlands;; t Centre for Advanced Cardiovascular Imaging, William Harvey Research Institute, Barts National Institute for Health Research Biomedical Research Centre, Queen Mary University of London, London, United Kingdom;; u Barts Heart Centre, St Bartholomew’s Hospital, Barts Health National Health Service Trust, West Smithfield, London, United Kingdom;; v Department of Radiology, Medical Center-University of Freiburg, Faculty of Medicine, University of Freiburg, Freiburg, Germany;; w Stanford University School of Medicine and Cardiovascular Institute, Stanford, California, USA.

**Keywords:** computed tomography angiography, coronary artery disease, fractional flow reserve, invasive coronary angiography, myocardial ischemia, myocardial perfusion imaging

## Abstract

**OBJECTIVES:**

In this international, multicenter study, using third-generation dual-source computed tomography (CT), we investigated the diagnostic performance of dynamic stress CT myocardial perfusion imaging (CT-MPI) in addition to coronary CT angiography (CTA) compared to invasive coronary angiography (ICA) and invasive fractional flow reserve (FFR).

**BACKGROUND:**

CT-MPI combined with coronary CTA integrates coronary artery anatomy with inducible myocardial ischemia, showing promising results for the diagnosis of hemodynamically significant coronary artery disease in single-center studies.

**METHODS:**

At 9 centers in Europe, Japan, and the United States, 132 patients scheduled for ICA were enrolled; 114 patients successfully completed coronary CTA, adenosine-stress dynamic CT-MPI, and ICA. Invasive FFR was performed in vessels with 25% to 90% stenosis. Data were analyzed by independent core laboratories. For the primary analysis, for each coronary artery the presence of hemodynamically significant obstruction was interpreted by coronary CTA with CT-MPI compared to coronary CTA alone, using an FFR of ≤0.80 and angiographic severity as reference. Territorial absolute myocardial blood flow (MBF) and relative MBF were compared using C-statistics.

**RESULTS:**

ICA and FFR identified hemodynamically significant stenoses in 74 of 289 coronary vessels (26%). Coronary CTA with ≥50% stenosis demonstrated a per-vessel sensitivity, specificity, and accuracy for the detection of hemodynamically significant stenosis of 96% (95% CI: 91–100), 72% (95% CI: 66–78), and 78% (95% CI: 73–83), respectively. Coronary CTA with CT-MPI showed a lower sensitivity (84%; 95% CI: 75–92) but higher specificity (89%; 95% CI: 85–93) and accuracy (88%; 95% CI: 84–92). The areas under the receiver-operating characteristic curve of absolute MBF and relative MBF were 0.79 (95% CI: 0.71–0.86) and 0.82 (95% CI: 0.74–0.88), respectively. The median dose-length product of CT-MPI and coronary CTA were 313 mGy·cm and 138 mGy·cm, respectively.

**CONCLUSIONS:**

Dynamic CT-MPI offers incremental diagnostic value over coronary CTA alone for the identification of hemodynamically significant coronary artery disease. Generalized results from this multicenter study encourage broader consideration of dynamic CT-MPI in clinical practice. (Dynamic Stress Perfusion CT for Detection of Inducible Myocardial Ischemia [SPECIFIC]; NCT02810795)

Coronary computed tomography (CT) angiography (CTA) has changed the practice of cardiovascular medicine by effectively ruling out coronary artery disease (CAD) in various clinical settings. However, coronary CTA tends to overestimate angiographic severity, and it cannot measure functional significance (1,2). Hence, clinical management decisions often require further functional testing (3). New techniques such as CT-derived fractional flow reserve (CTFFR) and CT myocardial perfusion imaging (CT-MPI) may address this limitation (4,5). Dynamic CT-MPI can quantify myocardial blood flow (MBF) during pharmacologic hyperemia from the myocardial enhancement patterns after injection of contrast medium (6). Absolute measures of MBF offer potential advantages to quantify the ischemia severity and identify balanced ischemia. Despite favorable diagnostic performance in single-center studies (5,7,8), CT-MPI has not yet found widespread clinical use because of the relative complexity of the test and radiation exposure. The latest-generation CT scanners offer higher spatial and temporal resolution with wider detector arrays and lower radiation doses, providing a more effective imaging approach (9,10). This prospective international multicenter study aims to evaluate the diagnostic performance of dynamic CT-MPI in addition to coronary CTA by using third-generation dual-source CT compared to invasive coronary angiography (ICA) and invasive FFR as the reference standard.

## METHODS

### STUDY DESIGN.

The SPECIFIC (Dynamic Stress Perfusion CT for Detection of Inducible Myocardial Ischemia) study is an international, multicenter, prospective, observational cohort study designed to investigate the diagnostic accuracy of dynamic CT-MPI (NCT02810795). Study participants were recruited at 9 hospitals in Europe, Japan, and the United States. The study protocol was compliant with the Declaration of Helsinki and received approval from the research ethics committee at each institution. All participants provided written informed consent.

### STUDY POPULATION.

Symptomatic patients aged ≥21 years with suspected stable CAD and scheduled for ICA were eligible for the study. Study exclusion criteria were as follows: 1) hemodynamically unstable condition, 2) prior myocardial infarction, 3) coronary bypass surgery, 4) percutaneous coronary intervention for myocardial infarction, 5) significant other cardiovascular diseases affecting CT-MPI performance (eg, heart failure, severe valvular regurgitation), 6) estimated glomerular filtration rate of <60 mL/kg/min, 7) body mass index of >35 kg/m^2^, 8) atrial fibrillation or other significant arrhythmias (>6 ectopic beats/min), 9) allergy to iodinated contrast medium, 10) pregnancy, and 11) contraindications to adenosine. Patients were excluded from the analysis if CT-MPI, coronary CTA, or ICA was not performed.

### IMAGING PROTOCOL.

#### Patient preparation.

**FIGURE 1:**
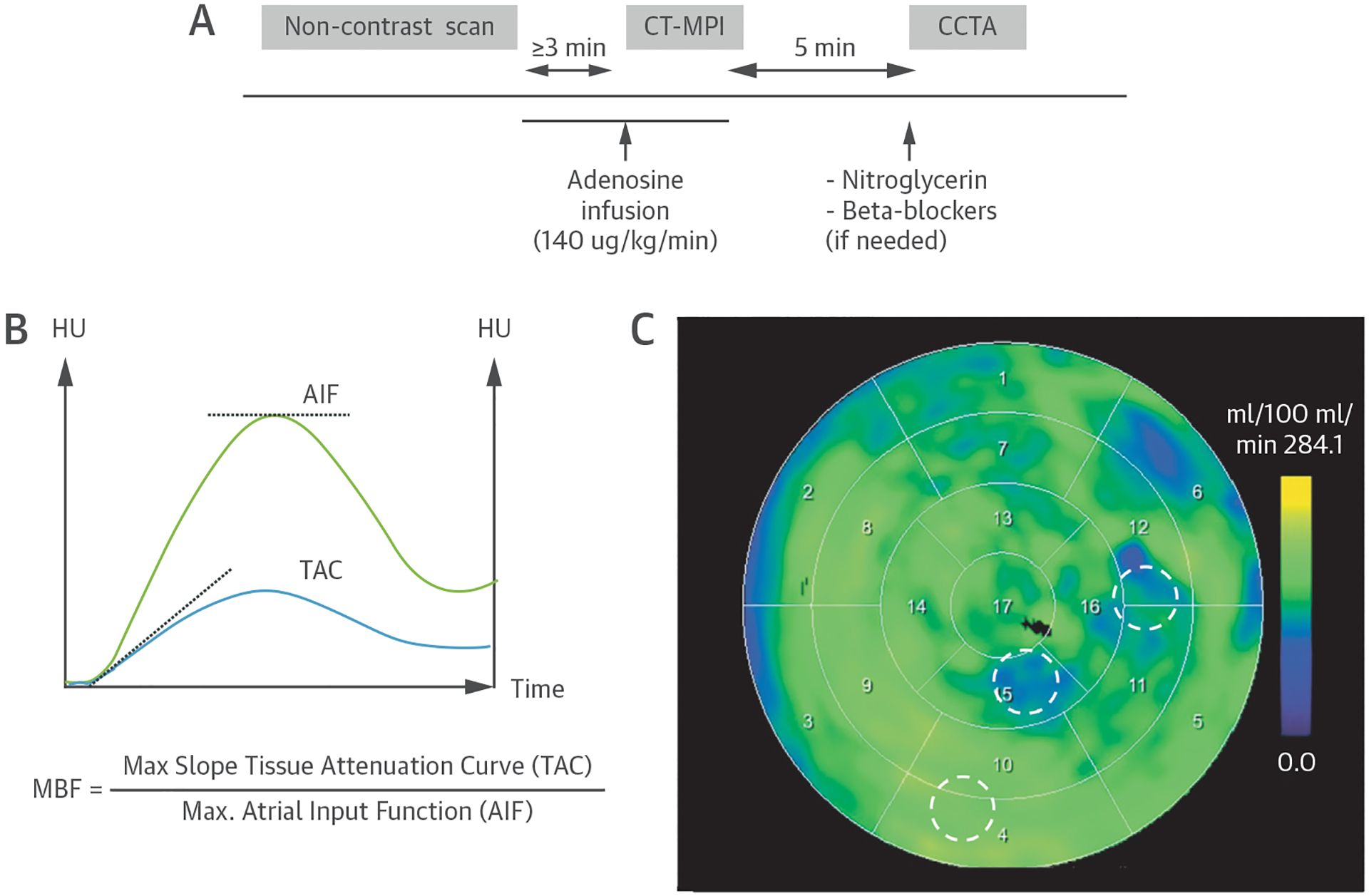
Study Protocol and Analysis **(A)** Dynamic stress CT-MPI and coronary CTA study protocol. **(B)** CT-MPI postprocessing: AIF curve and TAC to calculate MBF. **(C)** CT-MPI analysis: volumes of interest **(circles)** placed on a color-coded polar map. AIF = atrial input functional; CT-MPI = computed tomography myocardial perfusion imaging; CTA = computed tomography angiography; CCTA = coronary computed tomography angiography; HU = Hounsfield units; MBF = myocardial blood flow; TAC = time-attenuation curve.

Patients underwent a noncontrast scan, followed by CT-MPI, and coronary CTA on a third-generation dual-source CT scanner (SOMATOM Force, Siemens Healthineers) (Figure 1A). Patients were asked to refrain from caffeine-containing beverages for 12 hours and nicotine for 3 hours before the examination. Sublingual nitroglycerin was given before coronary CTA, as well as intravenous beta-blockers if the heart rate was >75 beats/min.

#### Dynamic stress CT-MPI.

Hyperemia was induced by intravenous adenosine (140 mg/kg/min) over ≥3 minutes. The standard contrast injection protocol was a 45-mL contrast bolus at 5.5 mL/s (iopromide, Bayer) (370 mg/mL), followed by 40 mL saline, with minor modification at 2 sites because of availability. The CT-MPI scan started 4 seconds after contrast injection, using alternating table positions (shuttle mode) for complete myocardial coverage. The data set consisted of 10 to 15 CT data samples over 30 seconds. The cardiac rhythm was continuously monitored, and the blood pressure was measured at regular intervals. The CT-MPI scan parameters were as follows: 2 × 96 × 0.6- mm collimation resulting in a 105-mm *z*-axis coverage by shuttle mode, 250-ms gantry rotation time, 66-ms temporal resolution, and tube voltage of 70 to 80 kV using the automated exposure control (300 mAs/rotation at 80 kV as reference). The 3.0-mm-thick slices were reconstructed with 2.0-mm overlap. CT-MPI data were evaluated at an independent core laboratory (Centre of Advanced Cardiovascular Imaging, Barts Cardiovascular Biomedical Research Center, London, United Kingdom). Image quality was assessed using a 4-point Likert scale. CT-MPI images with poor image quality were excluded from the analysis.

#### Coronary CTA.

Coronary CTA scan was acquired 5 minutes after CT-MPI using prospective electrocardiogram-triggered axial or high-pitch spiral scans. Tube current and voltage were (semi-)automatically selected based on body size. Scan timing was determined with a 10-mL contrast test bolus plus 40 mL saline or using bolus tracking. For coronary CTA, the contrast volume was 65 (interquartile range [IQR]: 55- 75) mL, injected at 5.0 (IQR: 4.9–5.4) mL/s with a 40-mL saline bolus chaser. Images were reconstructed with a medium-smooth kernel, 0.6-mm slice thickness, and 0.4-mm increment. For 34 patients, adequate-quality coronary CTA was clinically performed within 4 months of study enrollment. In these patients, the research coronary CTA was not performed. The coronary CTA data were transferred to a coronary CTA core laboratory (University of Tubingen, Tubingen, Germany). Coronary CTA images assessed as poor quality (6 vessels in 4 patients) were not excluded but were classified as positive for obstructive CAD.

#### CT-MPI data postprocessing.

CT-MPI source images were processed using commercial software (Syngo.CT Myocardial Perfusion, Siemens Healthineers). A motion correction algorithm was applied to align the serial samples. The left ventricular myocardium was isolated using a method of blood pool removal based on attenuation value thresholds. The arterial input function was derived from attenuation values measured in the descending aorta sampled in both dynamic image stacks. Time-attenuation curves were created for each myocardial volumetric element (voxel) within the left ventricle volume of interest. Dedicated parametric deconvolution based on a 2-compartment model of intra- and extravascular space was applied to fit the time-attenuation curves and compute MBF (11). MBF was calculated as the ratio between the maximum slope of the fit curve and the peak arterial input function (Figure 1B). The data were then processed using prototype software (Cardiac Functional Analysis Prototype, Siemens Healthineers) to automatically segment the left ventricle based on a heart model (12) and generate 17-segment polar maps representing the MBF distribution within the subendocardial layer of the left ventricular myocardium (Figure 1C) (13).

#### Integration of coronary CTA and CT-MPI data.

A comprehensive coronary CTA and CT-MPI core laboratory reading was performed by Christoph Artzner (coronary CTA core laboratory), Francesca Pugliese (CT-MPI core laboratory), and Koen Nieman (principal investigator) to visually match the coronary anatomy with the subtended myocardial territories and assess myocardial hypoperfusion per coronary branch. The readers were blinded to the ICA and FFR results. First, coronary stenoses were classified per vessel following Society of Cardiovascular Computed Tomography criteria (14). Second, CT-MPI maps were used for side-by-side comparison to the coronary CTA images. The patient’s coronary anatomy on coronary CTA was used to assign myocardial perfusion defects to specific coronary vessels. Based on the interpretation of available coronary CTA and CT-MPI images, the presence of hemodynamically significant CAD was determined per vessel territory. If coronary CTA and CT-MPI findings were discordant, then myocardial perfusion overruled coronary CTA stenosis severity, unless CT-MPI image quality was compromised. The most severely affected coronary branch determined per-territory disease classification. To calculate MBF per-vessel territory, a region of interest (corresponding to ≥0.5 cm^3^ of subendocardial myocardium) was sampled onto the MBF polar maps for each vessel territory, either in the area of suspected ischemia or centrally within territories without suspected ischemia. The reference MBF was defined as the 75th percentile of the automatically generated global endocardial MBF, which represents a robust measure of normal MBF in a specific patient and a specific examination that is relatively unaffected by territorial ischemia or artifacts (15). The relative MBF was calculated per vessel territory as the absolute MBF divided by the reference MBF.

#### ICA and FFR.

ICA was performed following local standards with a median of 2 days (IQR: 1–6 days) after CT-MPI and 3 days (IQR: 1–23 days) after coronary CTA. By protocol, intermediate coronary lesions with visual diameter stenoses of 25% to 90% were interrogated by FFR, if considered technically feasible and safe by the operator. An FFR pressure wire was positioned distal to the stenosis of interest, after which hyperemia was induced by intravenous adenosine at 140 mg/kg/min. ICA images and FFR data were evaluated by an ICA core laboratory (Erasmus Medical Center, Rotterdam, the Netherlands) and an FFR core laboratory (University Medical Center Groningen, Groningen, the Netherlands) for independent reading, blinded to the CT findings. Quantitative coronary angiography (QCA) software (Caas, Pie Medical Imaging) was used to measure the angiographic stenosis severity in all coronary segments with a diameter of >1.5 mm. Hemodynamically significant CAD was defined as an FFR of ≤0.80, or angiographic stenosis severity of >90% if FFR could not be performed. The absence of hemodynamically significant disease was defined as an FFR of >0.80, or angiographic stenosis of <25% if FFR was not performed. Numerous studies have demonstrated that visual interpretation overestimates tight stenoses and underestimates mild stenoses when compared to QCA (16). In addition, a threshold of 70% stenosis by QCA has shown a 98% specificity for the presence of FFR-positive CAD (17). Therefore, very severe stenosis (>90%) or the absence of stenosis (<25%) interpreted by the clinical operators at the time of the catheterization required QCA confirmation by the ICA core laboratory of at least >70% stenosis or <40% stenosis, respectively. Vessels with intermediate stenosis and no FFR were excluded from the analysis.

### STATISTICAL ANALYSIS.

Continuous variables are presented as mean ± SD or median with IQR and categorical variables are given as frequencies and percentages. For the primary analysis, we evaluated the diagnostic performance of CT-MPI with coronary CTA to identify hemodynamically significant CAD on a per-vessel and per-patient level, as defined by invasive FFR. The diagnostic performances for ≥50% stenosis and ≥70% stenosis on coronary CTA alone and coronary CTA combined with qualitative perfusion defects on CT-MPI were reported as sensitivity, specificity, positive predictive value (PPV), negative predictive value (NPV), and accuracy with the 95% CI, with the ICA and FFR as reference. Diagnostic accuracy was defined as a proportion of accurate test results over the total test results. Sensitivity, specificity, and accuracy of coronary CTA and coronary CTA plus CT-MPI were compared using the McNemar test and PPV and NPV using the weighted generalized score statistic. To identify patients who will benefit most from an additional CT-MPI, the diagnostic accuracy was stratified by stenosis grading on coronary CTA (0%–25%, 25%–49%, 50%–69%, 70%–100%). Area under the receiver-operating characteristic curves (AUCs) were determined for absolute MBF and relative MBF and compared with C-statistics using the method of DeLong et al with FFR as the reference (18). The optimal cutoff values for absolute and relative MBF were identified using the Youden index. The association between the perfusion parameters and FFR was evaluated using Spearman correlation when both variables were not normally distributed. Differences in median perfusion parameters among the 5 FFR ranges (≤0.75, 0.76–0.80, 0.81–0.85, 0.86–0.90, ≥0.91) were tested using the Kruskal-Wallis test and Mann-Whitney *U* test. Differences in image quality of dynamic CT-MPI between experienced (>50 scans) and inexperienced centers (≤15 scans) were tested using the chi-square test. Based on a predicted rate of 1.5 stenosed vessels per patient and a 50% functionally significance rate by FFR, we determined that 120 cases would result in a sensitivity and specificity with acceptably narrow CIs (<0.15). Statistical analyses were performed using SPSS version 25 (IBM Corp) and R (R Core Team 2019, version 3.6.2, DTComPair package). Med-Calc version 19.5.3 (MedCalc Software) was used to compare the AUC. A *P* value of <0.05 was considered statistically significant.

## RESULTS

### STUDY POPULATION.

**FIGURE 2:**
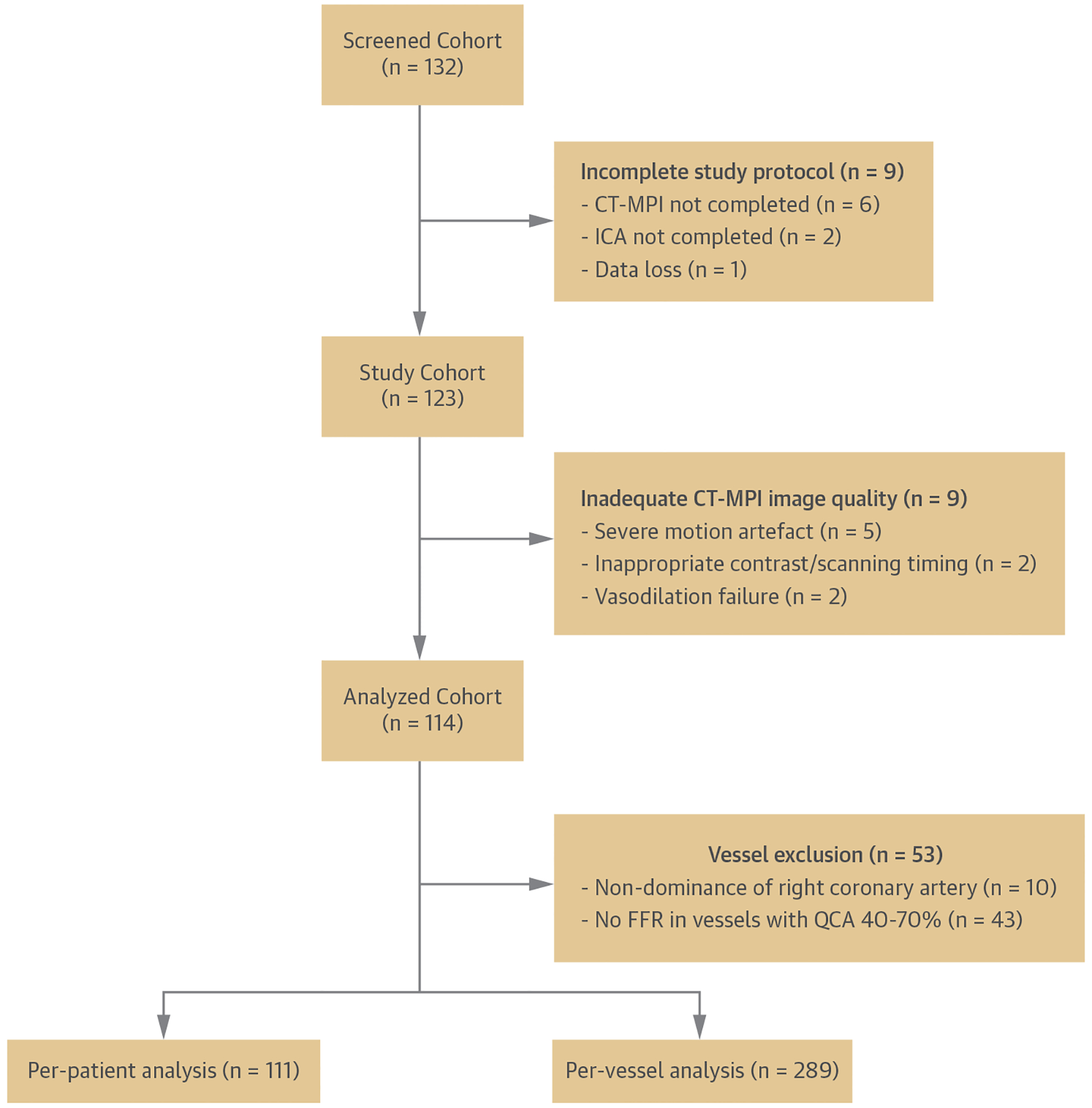
Inclusion Flowchart Study inclusion flowchart. CT-MPI = computed tomography myocardial perfusion imaging; FFR = fractional flow reserve; ICA = invasive coronary angiography; QCA = quantitative coronary angiography.

Between July 2016 and September 2019, 132 patients were enrolled, of whom 123 completed all examinations (Figure 2). No severe cardiac events or study-related complications were encountered during CT-MPI and invasive FFR. Image quality of CT-MPI was adequate for analysis in 114 patients (93%). The mean age was 64 ± 8 years, 66% were men, 39 (34%) patients had typical angina symptoms, and 5 (5%) patients had previously undergone stenting for stable CAD (Table 1). After exclusion of 53 vessels with indeterminate hemodynamically significance of disease by ICA/FFR, 289 coronary territories in 111 patients were available for the primary analysis. Functionally significant stenosis was present in 74 vessels (26%) and 54 patients (49%) based on an FFR of ≤0.80 (n = 54) or very severe angiographic stenosis (n = 20). Of these, 37 (33%) had single-vessel, 14 (13%) had 2-vessel, and 3 (3%) had 3-vessel disease of hemodynamic significance. Functionally significant stenosis was absent in 215 (74%) vessels and 57 (51%) patients based on an FFR of >0.80 (n = 74) or absent angiographic stenosis (n = 141). The median dose-length products of CT-MPI and coronary CTA were 313 mGy·cm (IQR: 237–448) and 138 mGy·cm (IQR: 76–280), respectively.

### DIAGNOSTIC PERFORMANCE OF CORONARY CTA AND CT-MPI.

**FIGURE 3:**
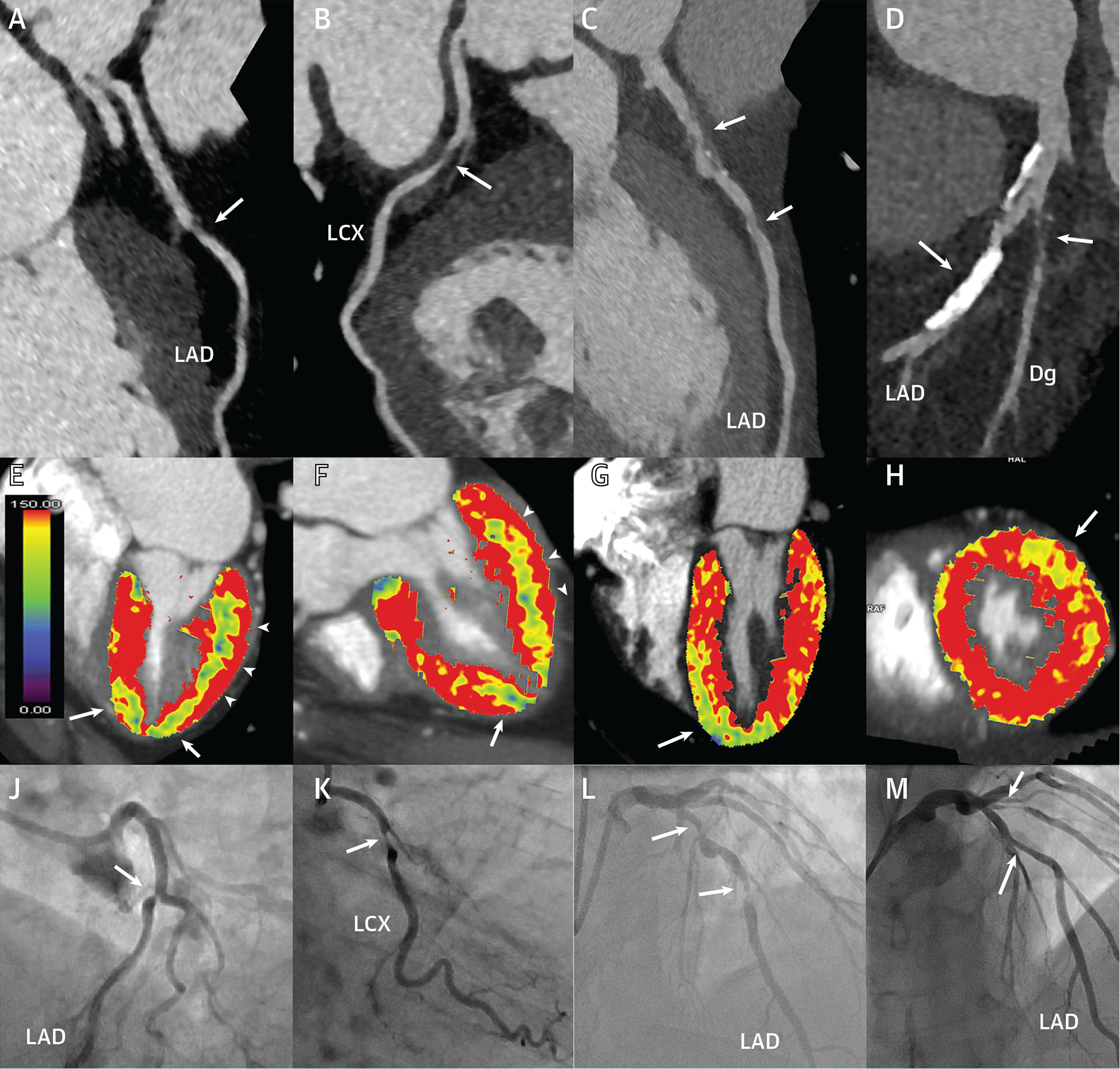
Case Examples Case 1: **(A)** Discrete narrowing in the LAD on CT (coronary CTA, **arrows**) and **(E, F)** an apical defect by perfusion imaging (CT-MPI, **arrows**) with **(J)** concordant ICA and an FFR of 0.76. The **color bar** in **A** displays the myocardial blood flow range from normal **(red)** to low **(green and blue)**. **(B)** The same patient had a second stenosis in the LCX, with **(E, F)** a posterolateral perfusion defect **(arrowheads)**, concordant with **(K)** ICA and FFR of 0.74. Case 2: **(C)** Diffuse, partially calcified narrowing and focal dilatation in the LAD on coronary CTA and a **(G)** CT-MPI perfusion defect in the distal septum and apex, confirmed by **(L)** ICA and an FFR of 0.56. Case 3: **(D)** Coronary CTA shows severely calcified plaque of uncertain angiographic stenosis severity in the LAD and a predominantly noncalcified severe stenosis in a large Dg. **(H)** There is a distinct anterolateral perfusion defect subtended by the Dg **(arrow)** but normal blood flow in the LAD territory. **(M)** ICA confirms the severe Dg stenosis (FFR: 0.68) and functionally nonsignificant, moderate mid-LAD stenosis (FFR: 0.83). CT = computed tomography; CT-MPI = computed tomography myocardial perfusion imaging; CTA = computed tomography angiography; Dg = diagonal branch; FFR = fractional flow reserve; ICA = invasive coronary angiography; LAD = left anterior descending coronary artery; LCX = left circumflex coronary artery.

Coronary CTA showed coronary calcium in 95 (83%) patients, stenoses of ≥50% in 131 vessels in 78 patients, and stenoses of ≥70% in 45 vessels in 37 patients. CT-MPI showed 85 ischemic territories in 60 patients (Figure 3). Coronary CTA with stenosis of ≥50% demonstrated a per-vessel sensitivity, specificity, PPV, NPV, and accuracy for the detection of hemodynamically significant stenosis of 96% (95% CI: 91–100), 72% (95% CI: 66–78), 54% (95% CI: 46–63), 98% (95% CI: 96–100), and 78% (95% CI: 73–83), respectively (Table 2 and Central Illustration). Stenosis of ≥70% on coronary CTA demonstrated higher specificity (94% vs 72%) but lower sensitivity (45% vs 96%) for the detection of hemodynamically significant stenosis. Coronary CTA with CT-MPI demonstrated a per-vessel sensitivity, specificity, PPV, NPV, and accuracy for the detection of hemodynamically significant stenosis of 84% (95% CI: 75–92), 89% (95% CI: 85–93), 73% (95% CI: 63–83), 94% (95% CI: 91–97), and 88% (95% CI: 84–92), respectively. Coronary CTA with CT-MPI demonstrated a higher specificity than coronary CTA stenosis of ≥50% (89% vs 72%; *P* < 0.001) but lower specificity than coronary CTA with stenosis of ≥70% (94%; *P* < 0.05). However, the sensitivity of coronary CTA with CT-MPI was higher than that of coronary CTA for stenosis of ≥70% (84% vs 45%; *P* < 0.001) but lower than that of coronary CTA for stenosis of ≥50% (96%; *P* < 0.01). Overall, the accuracy of coronary CTA with CT-MPI was higher than coronary CTA for stenosis of ≥50% and ≥70% (88% vs 78%; *P* < 0.001 and 82%; *P* < 0.05). In addition, on a per-patient level, the accuracy of coronary CTA with CT-MPI (84%; 95% CI: 77–91) was higher than for coronary CTA alone (73%; 95% CI: 65–81; *P* < 0.01 for ≥50% stenosis and 74%; 95% CI: 66–82; *P* = 0.07 for ≥70% stenosis). When stratified by stenosis grading on coronary CTA, the per-vessel diagnostic accuracy of coronary CTA stenosis ≥50% alone was 99% (95% CI: 98–100) for stenosis of 0% to 25%, 90% (95% CI: 76–100) for stenosis of 25% to 49%, 44% (95% CI: 33–55) for stenosis of 50% to 69%, and 84% (95% CI: 72–96) for stenosis of 70% to 100%. The diagnostic accuracy improved with the addition of CT-MPI in vessels with a stenosis between 50% and 69% from 44% (95% CI: 33–55) to 71% (95% CI: 61–81). No differences in overall accuracy were observed in other categories.

### QUANTITATIVE MBF ANALYSIS.

**FIGURE 4:**
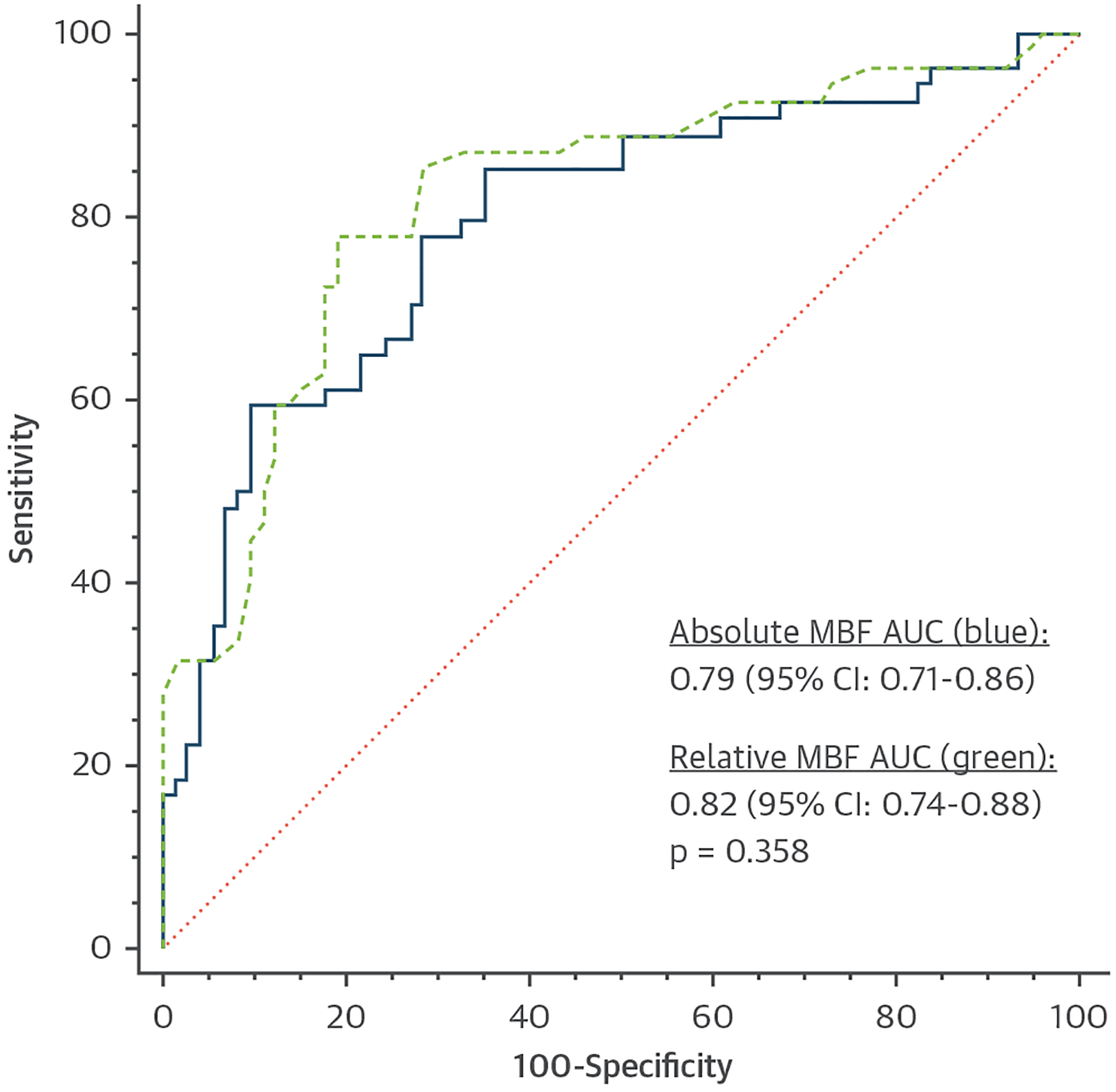
Receiver-Operating Characteristic Curves for Identifying Hemodynamically Significant Coronary Artery Disease Per-territory analysis with fractional flow reserve as the reference demonstrates similar AUCs for absolute **(blue)** and relative MBF **(green)**. AUC = area under the curve; MBF = myocardial blood flow.

**FIGURE 5:**
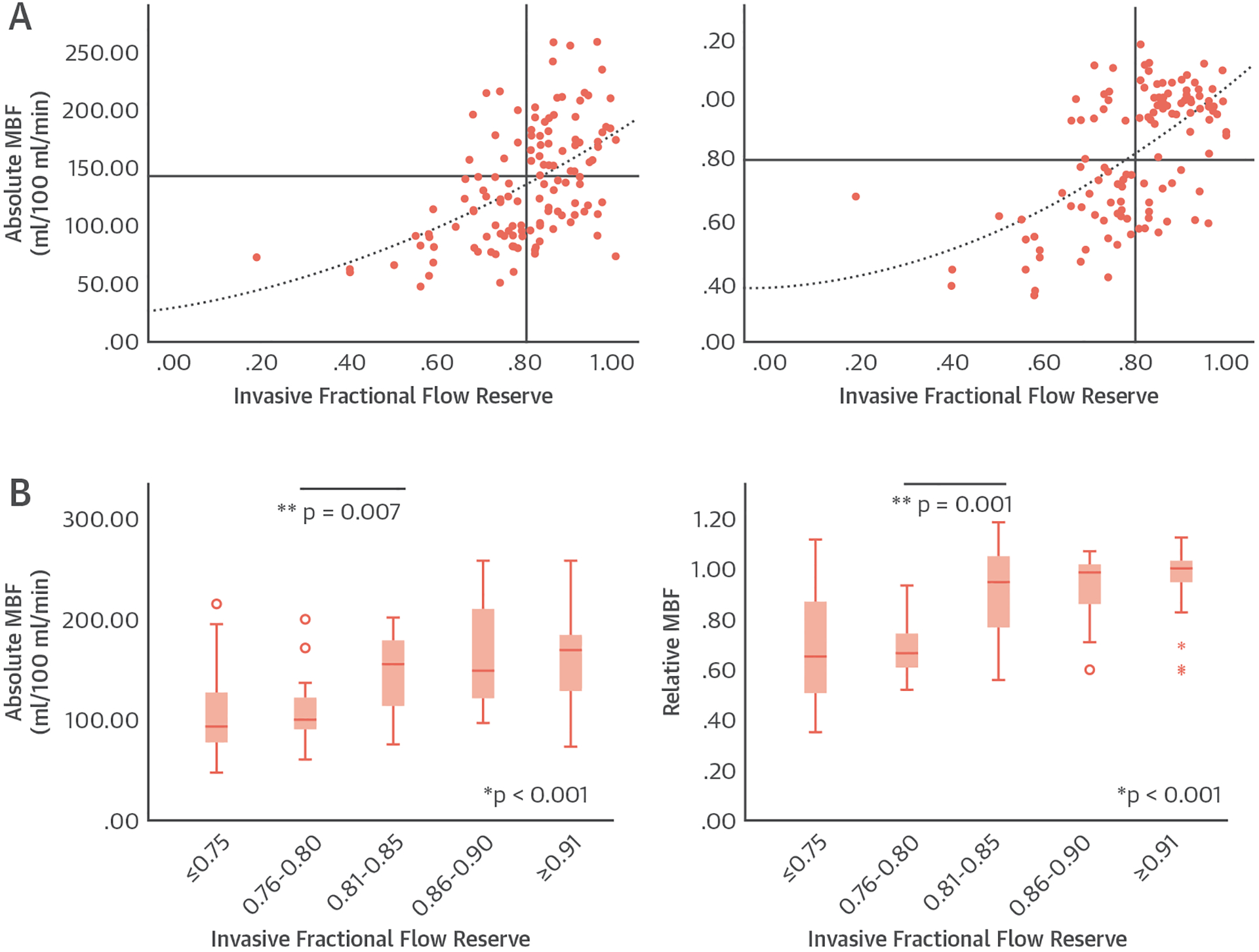
Correlation Between MBF and Invasive FFR Scatterplots comparing absolute **(left)** and relative **(right)** MBF with FFR with a correlation of 0.51 and 0.53, respectively. Horizontal and vertical lines are placed at the cutoff values of absolute MBF, relative MBF, and FFR. **(B)** Boxplots show median values (interquartile ranges) of absolute **(left)** and relative MBF **(right)**. *P value from Kruskal-Wallis test. **P value from Mann-Whitney *U* test. FFR = fractional flow reserve; MBF = myocardial blood flow.

The median absolute MBF was 97 mL/100 mL/min (IQR: 81–126 mL/100 mL/min) for territories supplied by vessels with functionally significant CAD and 158 mL/100 mL/min (IQR: 119–184 mL/100 mL/min) for remote territories (*P* < 0.001). The median relative MBF was 0.66 (IQR: 0.54–0.78) for functionally significant territories and 0.98 (IQR: 0.89–1.00) for remote territories (*P* < 0.001). The optimal thresholds for absolute MBF and relative MBF to identify functional significance were 142 mL/100 mL/min and 0.80, respectively. The AUCs of absolute MBF and relative MBF were 0.79 (95% CI: 0.71–0.86) and 0.82 (95% CI: 0.74–0.88), respectively (Figure 4). The Spearman correlation coefficients of absolute MBF and relative MBF with FFR were 0.51 and 0.53, respectively (*P* < 0.01) (Figure 5A). Both perfusion parameters were significantly lower in vessels with an FFR value <0.80 (*P* < 0.05) (Figure 5B).

### CT-MPI EXPERIENCE AND IMAGE QUALITY.

Four hospitals had previous dynamic CT-MPI experience (>50 scans), and 5 hospitals had no or limited dynamic CT-MPI experience (≤15 scans). CT-MPI was successfully completed in 64 of 68 (97%) patients enrolled by experienced centers and in 60 of 64 (94%) patients enrolled by centers without or with limited CT-MPI experience. The image quality was similar in experienced and inexperienced centers and showed a low prevalence of inadequate image quality: 6% and 8%, respectively (Table 3).

## DISCUSSION

### MAIN FINDINGS.

The main findings of this first international, multicenter study are that: 1) coronary CTA combined with dynamic CT-MPI identifies hemodynamically significant CAD; 2) CT-MPI increases overall accuracy compared to coronary CTA alone, specifically in vessels of moderate angiographic stenosis severity; and 3) absolute and relative MBF show no differences in differentiating territories with functionally significant CAD. This multicenter study confirms the incremental diagnostic value of dynamic CT-MPI, as reported in smaller, single-center studies.

### DIAGNOSTIC PERFORMANCE OF CT-MPI.

In a pooled analysis of single-center studies by Lu et al (19), dynamic CT-MPI with coronary CTA identified hemodynamically significant CAD with a sensitivity and specificity of 83% and 83%, respectively, compared to 82% and 61% by coronary CTA alone. In the present multicenter study with independent core laboratory analyses, comparable diagnostic performance was achieved with incremental value over coronary CTA alone. This study included centers with a range of prior CT-MPI experience but comparable technical performance, providing encouragement for broader clinical implementation. Invasive FFR and MPI are both functional tests, but each is based on different physiologic principles. Because of these mechanistic differences, even a perfect perfusion test could not be expected to exactly match the pressure drop over an epicardial stenosis in every single patient. Coronary CTA at a low stenosis threshold is very sensitive but not very specific. Therefore, it is virtually unavoidable that the addition of CT-MPI, or other functional tests that improve specificity and overall accuracy, will underestimate a number of lesions with an FFR of ≤0.80.

### QUANTITATIVE MBF ANALYSIS.

Dynamic CT-MPI and calculation of absolute MBF can be helpful in multivessel disease with balanced ischemia or microvascular disease (8,15,20). However, a challenge for dynamic CT-MPI is cardiac motion and myocardial displacement during the long breath-hold (21). Consequently, reported MBF cutoff values that signify hemodynamic significance vary substantially, from 75 to 164 mL/min/100 mL among studies (5,7–9). Therefore, several studies showed that MBF values normalized to remote myocardium outperform absolute MBF values (20,22). However, more recent studies contradicted these findings (15,23), and also, in the present study, we observe no significant difference between absolute (AUC: 0.79) and relative MBF (AUC: 0.82). Because the cutoff values for relative MBF are more consistent among studies and CT-MPI techniques (range 0.71–0.81), including this study (0.80), this may be a more robust parameter than absolute MBF for dynamic CT-MPI interpretation in real-world clinical practice.

### CT-MPI COMPARED TO ESTABLISHED AND EMERGING ALTERNATIVES.

A meta-analysis by Pontone et al (24) reported that the diagnostic accuracy of CT-MPI is in the same range as cardiac magnetic resonance and positron emission tomography and perhaps better than single-photon emission CT and stress echocardiography. Head-to-head comparisons between techniques are rare, except for a single-center study of CT-MPI and cardiac magnetic resonance perfusion imaging that reported equivalent performance (25). Static CT-MPI and CT-FFR are alternative CT applications for functional assessment of CAD. In a meta-analysis, dynamic CT-MPI has a higher sensitivity (85% vs 72%) but lower specificity (81% vs 90%) compared to static CT-MPI; however, no large head-to-head comparison studies have been performed (26). CT-FFR has shown similar and complementary diagnostic performance in head-to-head comparisons with CT-MPI (23,27). Advantages of dynamic CT-MPI over other perfusion techniques are the high spatial resolution and complete coverage of the left ventricle, as well as the ability to correlate perfusion abnormalities with coronary CTA findings, thereby integrating anatomy and function in one examination. Disadvantages of dynamic CT-MPI include the use of contrast medium and radiation. However, the radiation exposure of CT-MPI by third-generation dual-source CT was 314 mGy·cm, nearly 50% less than prior studies using second-generation dual-source CT (range 588–675 mGy·cm) (5,7,8). Similar dose reductions have been reported with contemporary wide-array CT systems (9). These doses are similar or lower than routinely used alternatives such as single-photon emission CT and ICA. CT-MPI is one of several techniques for the functional interpretation of CAD, each of which has strengths and weaknesses in terms of performance, safety, and cost. The incremental value of functional testing, as well as the management of stable coronary disease per se, will remain a topic of debate in the foreseeable future.

### CLINICAL ROLE OF CT-MPI.

In this study, the incremental value of CT-MPI was predominantly observed in moderately stenosed vessels. In populations with a low disease prevalence, the routine performance of CT-MPI would not be justified, but it may be offered after a coronary CTA with obstructive disease. The CRESCENT (Comprehensive Cardiac CT Versus Exercise Testing in Suspected Coronary Artery Disease) II trial showed a higher yield of coronary disease with a class I indication for revascularization when CT-MPI was selectively performed in patients with obstructive disease on coronary CTA, in comparison to stress testing, without increasing overall catheterization rates (28). Yu et al (29) showed that the addition of CT-MPI decreased the rate of ICAs not followed by revascularization compared to coronary CTA alone. Based on various considerations and local context, multiple viable options exist to functionally assess CAD. If a coronary CTA of adequate quality is available, CT-FFR represents an attractive option without the need for further physical testing and associated risks. However, the proportion of coronary CTA scans of adequate quality for CT-FFR processing varies substantially among studies, and CT-FFR has not been validated in a range of clinical conditions (eg, stents, grafts, anomalous coronary anatomy). In patients with diffuse disease, CT-MPI integrated with coronary CTA can depict lesion-specific functional impact.

### STUDY LIMITATIONS.

MPI and pressure-wire–based FFR are fundamentally different approaches to determine functional CAD severity. FFR assesses the hemodynamic significance of epicardial coronary stenoses, whereas MPI reflects the combination of epicardial and microvascular disease. By study design, the CAD prevalence was relatively high in our cohort, and extrapolation of the results to populations with lower disease probabilities should be done with care. Furthermore, we did not adjust for potential correlation between multiple vessels in the same subject, which might have underestimated the SEs of our analyses. Additionally, absolute and relative MBF thresholds indicating myocardial ischemia vary among studies and may be affected by the type of CT scanner and postprocessing software. Thus, future investigation is warranted to confirm our results on different CT scanners and postprocessing software. Similar to prior studies, and for a range of practical reasons including discrepancies between visual and QCA stenosis severity, FFR was not performed in all vessels with an intermediate stenosis severity (30).

## CONCLUSIONS

Dynamic CT-MPI offers incremental diagnostic value over coronary CTA alone for the identification of hemodynamically significant CAD. Generalized results from this multicenter study encourage broader consideration of dynamic CT-MPI in clinical practice.

## Figures and Tables

**CENTRAL ILLUSTRATION:**
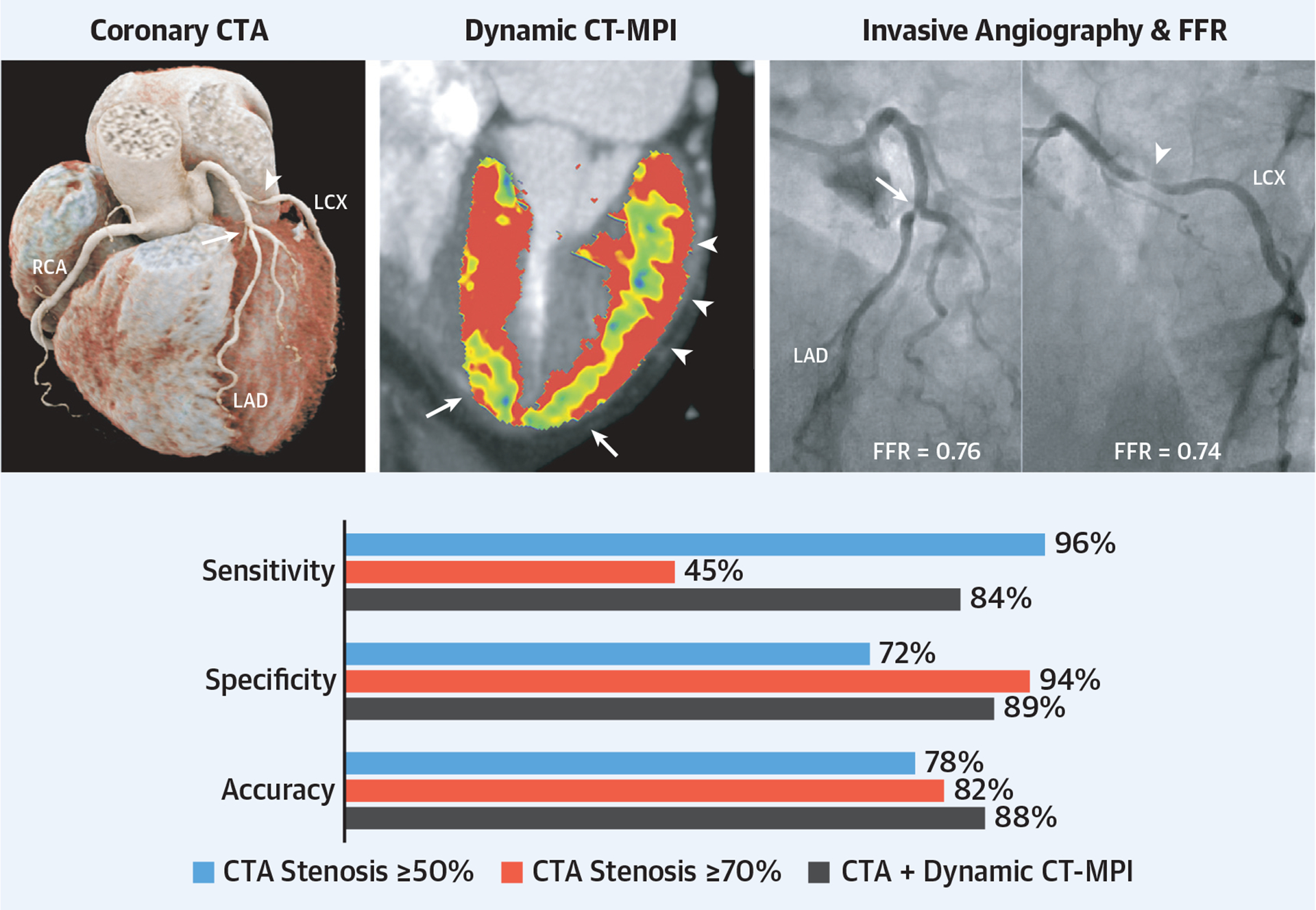
Diagnostic Accuracy of Computed Tomography Angiography and Dynamic Perfusion Computed Tomography for Hemodynamically Significant Coronary Artery Disease Coronary computed tomography angiography(CTA) and invasive angiography demonstrating moderate stenosis **(arrow)** in the left anterior descending coronary artery and severe stenosis **(arrowhead)** in the left circumflex coronary artery. Dynamic stress computed tomography myocardial perfusion imaging demonstrated corresponding perfusion defects **(yellow-blue)** in the apex and lateral wall, indicating inducible ischemia, as confirmed by fractional flow reserve. The bar graph below summarizes the diagnostic performance of CTA with a coronary stenosis threshold of 50% and 70% to CTA combined with perfusion imaging.

**TABLE 1:** Patient Demographics

Age, y	64 ± 8
Men	75 (66)
Body mass index, kg/m^2^	26 ± 4
Risk factors	
Current or previous smoker	61 (54)
Diabetes mellitus^a^	22 (19)
Dyslipidemia^a^	83 (73)
Hypertension^a^	81 (71)
Family history of coronary artery disease^b^	59 (52)
Previous percutaneous coronary intervention	5 (4)
Symptoms	
Typical angina	39 (34)
Atypical angina	35 (31)
nonanginal symptom	40 (35)
ICA and FFR	
Patients with coronary lesion causing ischemia, %^c^	54/111
Single-vessel disease, %	37 (33)
2-vessel disease, %	14 (13)
3-vessel disease,%	3 (3)
Number of vessels evaluated	289
Vessels with stenosis on QCA of ≥50%	84 (29)
Vessels with stenosis on QCA of ≥70%	29 (10)
Vessels with coronary lesion causing ischemia^c^	74 (26)
Right coronary artery	18 (6)
Left main/left anterior descending coronary artery	41 (14)
Left circumflex artery	15 (5)
CT-MPI	
Heart rate baseline, beats/min	66 (60–75)
Heart rate during adenosine stress, beats/min	83 (70–93)
Image quality	
Excellent	66 (58)
Good	39 (34)
Moderate	9 (8)
Dose-length product, mGy·cm	313 (237–448)
Coronary CTA	
Beta-blocker administered	37 (32)
Image quality	
Excellent	60 (53)
Good	40 (35)
Moderate	10 (9)
Poor	4 (4)
Dose-length product, mGy·cm	138 (76–280)

Values are n (%), mean ± SD, or median (interquartile range).

a Based on medication use.

b Family history of coronary artery disease having first- or second-degree relatives with premature coronary artery disease (age: 55 y).

c Functionally significant coronary lesion defined as FFR of ≤0.80 or visual diameter narrowing of ≥90% combined with a QCA of ≥70%.

CTA = computed tomography angiography; CT-MPI = computed tomography myocardial perfusion imaging; FFR = fractional flow reserve; ICA = invasive coronary angiography; QCA = quantitative coronary angiography.

**TABLE 2:** Diagnostic Performance of Coronary CTA and CT-MPI

	Sensitivity	Specificity	PPV	NPV	Accuracy	TP	TN	FP	FN
Per vessel									
Coronary CTA stenosis of ≥50%	96 (91–100)	72 (66–78)	54 (46–63)	98 (96–100)	78 (73–83)	71	155	60	3
Coronary CTA stenosis of ≥70%	45 (33–56)	94 (91–98)	73 (60–87)	83 (78–88)	82 (77–86)	33	203	12	41
Coronary CTA plus CT-MPI	84^a,b^ (75–92)	89^a,b^ (85–93)	73^a^ (63–83)	94^a,b^ (91–97)	88^a,b^ (84–92)	62	192	23	12
Per patient									
Coronary CTA stenosis of ≥50%	94 (88–100)	53 (39–66)	65 (55–76)	91 (81–100)	73 (65–81)	51	30	27	3
Coronary CTA stenosis of ≥70%	57 (44–71)	89 (81–98)	84 (71–96)	69 (58–80)	74 (66–82)	31	51	6	23
Coronary CTA plus CT-MPI	89^b^ (80–98)	79^a^ (68–90)	80^a^ (70–90)	88^b^ (79–97)	84^a^ (77–91)	48	45	12	6

Values are % (95% CI) or n. Sensitivity, specificity, and accuracy of coronary CTA stenosis of ≥50% and ≥70% were compared to coronary CTA plus CT-MPI using the McNemar test and PPV and NPV using the weighted generalized score statistic.

a *P* < 0.05 for coronary CTA stenosis ≥50%.

b *P* < 0.05 for coronary CTA stenosis of ≥70%.

FN = false negative; FP = false positive; NPV = negative predictive value; PPV = positive predictive value; TN = true negative; TP = true positive; other abbreviations as in Table 1.

**TABLE 3:** Image Quality By Dynamic Computed Tomography Myocardial Perfusion Imaging Experience

	Excellent	Good	Moderate	Poor
Experienced centers (>50 scans)	30 (45)	29 (44)	7 (11)	0 (0)
Inexperienced centers (≤15 scans)	37 (62)	15 (25)	6 (10)	2 (3)

Values are n (%). Differences in image quality were tested using the chi-square test *(P* = 0.072).

## PERSPECTIVES

### COMPETENCY IN MEDICAL KNOWLEDGE:

Dynamic CT-MPI with coronary CTA provided incremental diagnostic value over coronary CTA alone in patients with suspected CAD. This encourages broader considerations of dynamic CT-MPI in the clinical evaluation of patients with suspected CAD.

### TRANSLATIONAL OUTLOOK:

The potential role of coronary CTA with dynamic CT-MPI to provide interventional cardiologists and cardiac surgeons with an anatomy and functional noninvasive roadmap for the decision making of myocardial revascularization strategy needs to be investigated. Additionally, studies on different CT scanners and postprocessing software are needed to better understand how absolute and relative MBF can be used in clinical practice.

## ABBREVIATIONS AND ACRONYMS

AUC: area under the receiver-operating characteristic curve
CAD: coronary artery disease
CT: computed tomography
CTA: computed tomography angiography
CT-MPI: computed tomography myocardial perfusion imaging
FFR: fractional flow reserve
ICA: invasive coronary angiography
IQR: interquartile range
MBF: myocardial blood flow
NPV: negative predictive value
PPV: positive predictive value
QCA: quantitative coronary angiography
